# Public Preferences Regarding Equitable Healthcare Rationing Across Gender Identities in China

**DOI:** 10.3390/ijerph22081218

**Published:** 2025-08-04

**Authors:** Chau-kiu Cheung, Zenan Wu, Eileen Yuk-ha Tsang

**Affiliations:** Department of Social and Behavioural Sciences, City University of Hong Kong, Hong Kong, China

**Keywords:** public healthcare, rationing, affirmation, universalism, egalitarianism, equity, rationality, self-interest, public interest, personal contribution, social contribution

## Abstract

Public opinion on public healthcare rationing regarding gender identity is crucial for democratic policymaking because of public concern regarding sexual orientation, gender identity, and gender expression (SOGIE). Based on rationality theory, rationally equitable rationing depends on equity orientations and prioritizing public interest over self-interest. Specifically, equity orientations include those toward equality, need, personal contribution, and social contribution. To project public preference for public healthcare rationing, this study involved 744 Chinese people in a web survey. These participants indicated their preferences for public healthcare rationing and self-interest, public interest, and equity orientations, including those based on contribution, equality, and need. Regression analysis based on the rationality framework showed that public healthcare rationing that was equal across SOGIE identities was predominantly preferable, based on rational equity. In contrast, public healthcare rationing without considering SOGIE was less preferable, and rationing unequally across gender identities was not preferred. These results imply that affirmative and egalitarian rationing is the most rationally equitable approach.

## 1. Introduction

Projecting the rationally equitable rationing of public healthcare to people with diverse sexual orientation, gender identity, and gender expression (SOGIE) is crucial for policymaking. First, SOGIE diversity is a reality because people realize, emphasize, and act according to their identities [1]. Second, public healthcare is typically subject to resource scarcity [2]. Hence, public healthcare is likely subject to rationing in terms of the formal allocation of resources [3]. This rationing is expected to be equitable or fair [4], and acceptable equity needs to be based on rationality or sensible reasoning [5]. Rationally equitable rationing needs to follow public opinion to achieve democracy and ensure accountable and sustainable policymaking [6]. Being mostly uncharted, rational public opinion on equitable public healthcare rationing regarding people identifying with diverse SOGIE is the target of this study. 

Equitable public healthcare rationing is valuable, as equity is a social ideal or virtue [7]. Moreover, equity represents a social contract involving obligation and trust [8]. Therefore, equity is the basis for social functioning [7]. Equity also safeguards motivation and satisfaction and prevents anger and guilt [9]. Equitable rationing is instrumental in establishing legitimacy in policymaking [7], and it registers the virtue of sociotropy or concern for people and their conditions [10]. Rationing, in general, is necessary to distribute scarce and valued resources, such as public healthcare [11]. Public healthcare is vital and suitable for rationing because it hinges on professionalism and policymaking to secure efficiency and equity [12]. More generally, healthcare policymaking is indispensable to social welfare [13]. Public healthcare rationing is inevitable and prevalent, as in daily frontline discretion [14], and it is favorable for societal collaboration and cost-effectiveness [11]. This rationing is justifiable in light of public knowledge about healthcare and its needs and merit bases [11]. However, public healthcare rationing is controversial, questionable, or uncertain in terms of its rational and equitable grounds, such as discrimination [15]. Thus, it is open to rights violations [16]. This rationing is also unethical when it serves self-interest [11]. Given this controversy, unraveling rationally equitable rationing for public healthcare is imperative.

Rationing public healthcare to people either considers or ignores their SOGIE, corresponding to affirmative or universalist rationing, respectively. Affirmative rationing, which recognizes diverse SOGIE, is advisable for addressing problems because of these identities [17]. These problems are discrimination, exclusion, marginalization, rejection, stigmatization, and unfair treatment, triggering suffering, distress, and illness [18]. Hence, affirmative rationing means eliminating stigmatization and other problems because of SOGIE by affirming rather than concealing these identities. It can work in equal or egalitarian and unequal or prioritarian ways [19]. The former allocates healthcare to every SOGIE equally, whereas the latter allocates healthcare to SOGIE unequally, to prioritize some identities. In contrast, universalist rationing makes healthcare available to all who need it, regardless of their SOGIE, thereby dismissing SOGIE as a criterion for rationing [20]. Universalist rationing, therefore, acknowledges everybody’s rights, without bias or preferential treatment, and rests on social democracy, equality, or socialism for all [21]. Universalist rationing engenders equal rationing when people have equal needs.

Affirmative and universalist rationing follow or challenge equity bases or principles, including equality, merit, and need [7]. Equity based on equality means that everyone is equally worthy of receiving an equal share [22]. This equality criterion rests on religious grounds. In contrast, equity can also mean different treatment, depending on various conditions. Equity based on need highlights the view that a person in need deserves to receive care [7]. The need criterion or orientation reflects the ethic of care, compassion, responsibility, or sympathy [23]. Alternatively, equity based on merit, including personal and social achievements or contributions (i.e., contributions to society), upholds meritocracy [7]. This merit criterion or orientation builds on faith in people’s agency, education, mastery, perfectionism, competence, and striving [24]. Merit orientation also embodies personal and social responsibility [24].

Rationing is rational when embodying instrumental or substantive (or value-based) rationality [25]. These two forms of rationality support rationality theory [26]. This theory has evolved from the Weberian foundation [27]. On the one hand, instrumental rationality registers using certain means to achieve ends or goals based on personal or public interests, which are typically enjoyable or pleasurable. These interests include benefits or meeting specific needs or preferences, like health or wealth. Personal or self-interest reflects egocentrism, egoism, or selfishness [28]. Inherently, self-interest embraces entitlement, self-benefit, self-esteem, and self-evaluation [29], and it is ubiquitous because it is socially tolerable [30]. Alternatively, public interest builds on people’s involvement in society and is instrumentally rational due to societal reciprocity [31]. Therefore, public interest rests on a communal, organismic, or unitary view of society that is premised on complementarity and mutuality. Moreover, it prevails as civility or civic virtue [32]. On the other hand, substantive rationality demonstrates consonance with values, beliefs, or ideals that are not directly enjoyable or pleasurable. They include equity, liberty, loyalty, and religiosity. Equity is grounded on equality, merit, and need. Eventually, rationality theory posits that the two forms of rationality are responsible for rational rationing. The theory further envisions that rationally equitable rationing follows substantive rationality, based on the values of equity and its criteria.

Rationality theory is empirically valid in explaining and guiding opinions and actions. Hence, personal instrumental rationality or self-interest has dampened prosocial and preventive orientations and behaviors [33]. Self-interest has also lessened intimacy [34] and has fostered support for public provision and redistribution [35]. It has also underlain a sense of superiority and, thus, demands for privileges [36]. Like self-interest, public interest has reinforced public participation and the demand for public provision [37] and has also engendered support for democracy and social welfare [38]. Similarly, equity orientation has guided public participation, support for public spending and social welfare, and trust in government or public policy [39]. Particularly, equity orientation has eroded support for immigrants [40]. Equity orientation and its componential orientation to need have notably affected the preference for rationing [41]. This orientation on need has bred support for social welfare [42]. Equality orientation or egalitarianism has generated support for affirmative policies to ensure equality among explicit identities [43]. This orientation has affected agreement, demand, preference, and support regarding public policy and practice, such as in healthcare, redistribution, and social welfare [44]. Merit orientation justifies inequality [45] and has strengthened support for democracy or public involvement [46]. It has also safeguarded policy evaluation [47].

Rationality theory supports public healthcare rationing, affirmative policy, and universalist policy. Support for public healthcare rationing has stood on the substantive rationality of merit orientation [48]. Likewise, support for public healthcare reform arises from the substantive rationality of equity orientation [49]. More generally, support for public healthcare has stemmed from the substantive rationality of equality and need orientations [44]. Support for affirmative policy has rested on the instrumental rationality of self-interest [50]. This support has also stemmed from the substantive rationality of equity orientation and its componential orientations to equality and need orientations [43]. Support for universalist policy has its basis in the substantive rationality of equity orientation [51]. Support for universalist welfare has also rested on the instrumental rationality of self-interest [52].

Rationality theory thus provides an analytic framework to distill public preference for public healthcare rationing to achieve equity rationally. Accordingly, rationally equitable rationing rests on equity orientation, its componential orientations to equality, need, and merit, and public interest to fulfill substantive and publicly instrumental rationality. As such, rationing precludes interference from self-interest, background, and response characteristics. Background characteristics, such as gender, age, education, religious faith, marital status, and residential location, can affect public preference regarding healthcare [44,53]. Response characteristics, such as acquiescence and social desirability, can bias self-report ratings [54]. Removing all these biases is necessary to achieve rationally equitable rationing.

### China’s Context

China is worth studying in this regard because public healthcare rationing needs to be pertinent to the context [55]. Contextual pertinence and variation regarding SOGIEs are also noteworthy [56]. In China, gender diversity is the norm [57]. Nevertheless, although SOGIE and orientation are freely expressed, the non-LGBTQ+ form enjoys more approval, protection, and promotion than the LGBTQ+ form [58]. Accordingly, China’s current policy stipulates no explicit approval, disapproval, or promotion regarding SOGIE, while endorsing sexual rights [58]. Public attitudes toward SOGIE are traditionally conservative [58,59]. Consequently, those with LGBTQ+ identities suffer more arrests, discrimination, and punishment than those who do not [59]. This suffering obstructs SOGIE disclosure and authenticity [58]. Hence, affirmative policies are advisable for SOGIE [58]. Equality and merit orientations in public healthcare are prominent [55]. Alternatively, public interest can become predominant, thanks to collectivism [60]. Thus, equality orientation favors a universalist policy because of collectivist overtones [55]. Equity and instrumental rationality are not the most pressing concern [61]. These broad observations highlight the need for elucidating rationally equitable rationing. Public preferences are crucial in China because of the Chinese people’s propensity to conform to social norms regarding SOGIE [58].

## 2. Materials and Methods

This study relied on a web survey of 744 Chinese people (aged 18+ years) recruited by mainland Chinese graduate students in April and May 2025. These people met the criteria of being capable of and willing to complete the web survey independently. Possible respondents were approached personally via classmates (25.6%, Table 1), college forums (24.2%), emails (19.6%), and social media (30.4%). The respondents then indicated their consent to the anonymous and confidential survey, as approved by an institutional research ethics committee. The sample, comprising independent responses, could test a minimum effect size (*r* = 0.103) with 95% confidence and 80% statistical power (via SPSS).

### 2.1. Participants

Based on self-reporting, the survey sample comprised 59.0% identifying as female, 40.5% as male, 0.5% as another gender, 65.0% as heterosexual, 80.4% as irreligious, 55.2% in an urban residence, 54.5% with college or higher education, and 3.6% as married (Table 1). The average age was 23.9 years (*SD* = 4.9). Hence, the sample was relatively young and highly educated. 

### 2.2. Measurement

The survey presented questions within each section in different, randomly arranged orders to the different respondents to minimize the question order effect. These questions measured their preference for public healthcare rationing and orientations toward equity, equality, need, personal contribution, social contribution, self-interest, public interest, and social desirability. The questions included inversely scored items to minimize the response bias of acquiescence. Eventually, these questions generated scores on a 0–100 scale to facilitate comparison and interpretation.

Preference for public healthcare rationing (in the present) combined two questions: “Given limited public healthcare monetary resources, what should be the rationing of the resources to the following six persons simultaneously: lesbian, gay, bisexual, transgender, other than these four (i.e., cisgender and heterosexual), and queer or other?” and “Given limited public healthcare time resources, what should be the rationing of the resources to the following six persons simultaneously?” Preferences for each question were “equal” and “unequal” for affirmative rationing and “social orientation or gender identity or expression not a consideration” for universalist rationing. Eventually, the preferences combined from responses to the two questions were reliable: universalist rationing (α = 0.831, average factor loading = 0.925), unequal rationing (α = 0.764, average factor loading = 0.899), and equal rationing (α = 0.594, average factor loading = 0.843). 

Equity orientation in the previous three months combined three rating items, such as “emphasizing equity” [62]. It was reliable (α = 0.813).

Equality orientation in the previous month combined three rating items adapted to public healthcare, such as “Everyone should have equal public healthcare” [63]. It was reliable (α = 0.794).

Need orientation in the previous month, combined five rating items adapted to public healthcare, such as “More public healthcare should go to those with more serious illnesses” [63]. It was reliable (α = 0.886).

Personal contribution orientation in the previous month combined four rating items adapted to public healthcare, such as “Those who exercise regularly should get more public healthcare services” [63]. It was reliable (α = 0.854).

Social contribution orientation in the previous month combined six rating items adapted to public healthcare, such as “People who help their communities should receive extra public healthcare” [63]. It was reliable (α = 0.889).

Self-interest in the previous month combined four rating items adapted to public healthcare, such as “Public healthcare should satisfy my needs” [64]. It was reliable (α = 0.847).

Public interest in the previous month combined four rating items adapted to public healthcare, such as “Public healthcare should improve public health” [65]. It was reliable (α = 0.874).

Social desirability in the previous two months combined five rating items adapted to public healthcare, such as “Keeping promises” [66]. It was reliable (α = 0.688).

Acquiescence combined all rating items to indicate the tendency to rate everything highly. It was reliable (α = 0.960).

### 2.3. Analysis

Regression analysis revealed the bases of preferences for universalist, equal, and unequal public healthcare rationing regarding SOGIE, based on orientations and interests, background, and response characteristics. The orientations covered equity, equality, need, personal contribution, and social contribution, and the interests covered self- and public interest. The analysis entered predictors for three cumulative steps: background characteristics, equity orientation and response characteristics, and other orientations and interests. These bases then enabled the projection of preferences for rationally equitable rationing, based on maximum equity and its componential orientations and public interest and minimum self-interest, with background and response characteristics at their average levels.

## 3. Results

Regression analysis revealed that preferences for universalist, equal, and unequal rationing significantly rested on different bases, without a multicollinearity problem (VIF < 5.8). Specifically, a preference for universalist rationing hinged on public interest (*β* = 0.348, see Table 2) and declined with personal contribution orientation (*β* = −0.155) and equality orientation (*β* = −0.119). A preference for equal rationing affirmed that SOGIE increased with equality orientation (*β* = 0.110) and decreased with self-interest (*β* = −0.198). Alternatively, the preference for unequal rationing affirmed that SOGIE rose with self-interest (*β* = 0.209) and receded with equity orientation (*β* = −0.099). These preferences also significantly varied according to background and response characteristics, which were control factors in the analysis. Specifically, a preference for universalist rationing increased with education (*β* = 0.099), marriage (*β* = 0.180), and urban residence (*β* = 0.123). In contrast, a preference for equal rationing declined with marriage (*β* = −0.094) and urban residence (*β* = −0.111). This preference did not significantly vary by age or gender. Moreover, the preference for unequal rationing diminished with education (*β* = −0.108).

**Figure 1:**
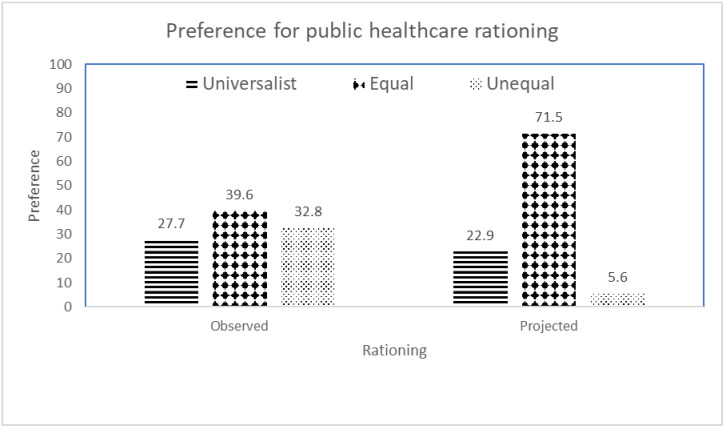
Observed and projected preferences for public healthcare rationing.

The regression analysis enabled the projection of rationally equitable preferences for public healthcare rationing, based on maximum equity orientations and public interest and minimum self-interest. This preference was especially high for equal rationing to affirm SOGIE (*M* = 71.5, Table 3 and Figure 1), low for universalist rationing (*M* = 22.9), and minimal for unequal rationing to affirm SOGIE (*M* = 5.6). Remarkably, these projected preferences dramatically contrasted with the observed preferences not projected for rational equity (*M* = 27.7–39.6, Table 1 and Figure 1). Without maximizing rational equity, the latter exhibited that the three ways of rationing tended to be equally preferable.

## 4. Discussion

Public preferences for public healthcare rationing rested on instrumental and substantive rationality, as envisioned by rationality theory. Specifically, the instrumental rationality of public interest buttressed the preference for universalist rationing and dismissed the preference for unequal rationing to affirm SOGIE. In contrast, the substantive rationality of equality orientation underpinned the preference for equal rationing to affirm SOGIE and eroded the preference for universalist rationing. The substantive rationality of equity orientation diminished the preference for unequal rationing. Moreover, the substantive rationality of personal contribution orientation dampened the preference for universalist rationing. Based on this rationalization, universalist rationing hinges on the instrumental rationality of public interest as opposed to the substantive rationality of personal contribution and equality. Equality rationing to affirm SOGIE builds on the substantive rationality of equality orientation as opposed to the instrumental rationality of self-interest. Conversely, unequal rationing to affirm SOGIE depends on the instrumental rationality of self-interest as opposed to the instrumental rationality of public interest and the substantive rationality of equity orientation.

Universalist public healthcare rationing is instrumental to the public interest and contradicts equality and personal contribution, based on rationality analysis. This instrumentality echoes the contributions of universalist policy to public livelihoods, such as employment and housing, as opposed to poverty [67]. This contribution rests on citizenship, cooperation, partnership, security, and solidarity, as embraced in universalist policy [68]. However, universalist rationing may not achieve equality when equality is not a criterion for rationing. As such, universal rationing maintains the status quo of inequality. Universalist rationing also contradicts personal contribution when such a contribution is not a rationing criterion. Accordingly, cooperation and partnership, rather than individual contribution, characterize universalist policy [68].

Universalist public healthcare rationing was more preferable for those with higher education, in a marriage, and with an urban residence. Education elevated the value of egalitarianism in treating all people impartially [69]. Hence, the contribution of education to the preference for universalist rationing reflects the substantive rationality posited in rationality theory. Marriage has weakened support for LGBTQ+ and affirmative policy [70]. Thus, marriage has engendered the preference for universalist rationing over affirmative rationing. This preference reflects the substantive rationality posited in rationality theory. Besides, marriage is less common among LGBTQ+ people [71], so married people would not benefit from affirmative rationing for LGBTQ+ individuals. The contribution of marriage to the preference for universalist rationing over affirmative rationing reflects the self-interest rationality of rationality theory. Having an urban residence raised healthcare use [72]. Healthcare use means receiving more benefits from universalist healthcare rationing for every user than the other systems. Therefore, an urban residence contributes to a preference for universalist healthcare rationing because of the self-interest rationality of rationality theory.

Equal public healthcare rationing to affirm SOGIE matches equality and impedes personal interests, according to rationality analysis. Essentially, equal rationing as an egalitarian policy downplays personal talent and, thus, individual interests [73]. When rationing shares out resources equally, it cannot satisfy more demanding individuals [74].

Unequal or prioritarian public healthcare rationing to affirm or emphasize SOGIE contradicts equity, impedes public interests, and serves personal interests, according to rationality analysis. This contradiction arises because SOGIE provides no reason for unequal or prioritarian rationing. Accordingly, differences in health issues due to SOGIE remain uncertain [17]. Hence, unequal rationing may result in discrimination and inequity. Discrimination has also created social exclusion and inequality, jeopardizing public interests [75]. In contrast, unequal rationing prioritizes some SOGIE identities to amplify their personal interests.

Equal public healthcare rationing to affirm SOGIE is predominantly preferable, given maximal equity orientations, maximal public interest, and minimal self-interest. This rationing maximizes equity and public interests and minimizes personal interests. Essentially, preferable rationing should be free of self-interest bias [11]. Hence, equal rationing is more rationally equitable than unequal or universalist rationing because of its egalitarianism and affirmation of SOGIE. On the one hand, egalitarianism is preferable because of its virtue of empathy [76] and because it is a simple, understandable norm [77]. Egalitarianism is also justifiable by or compatible with religion and other ideals [1]. Notably, egalitarianism is ideal for maximizing public health [78], safeguarding rights [1], and sustaining plurality [79]. In contrast, affirming SOGIE is preferable because of its demonstrated need [17]. This affirmation safeguards health [80] and diminishes discrimination, exclusion, inferiority, marginalization, and stigmatization [17]. Affirmative policy is preferable for sustaining diversity and social integration [81].

### 4.1. Limitations and Future Research

The self-report survey and its sample have limitations in terms of objectivity and representation. Accordingly, the survey relies on the respondents’ understanding of public healthcare rationing, equity, SOGIE, and various related factors. Self-reports based on such an understanding cannot be entirely objective, accurate, or verifiable. They reflect public opinion rather than scientific knowledge. Moreover, the sample recruited through Chinese graduate students was hardly representative of the population in China, not to mention the global one. Recruitment through students was selective and might introduce biases. Moreover, the sample was relatively young and college-affiliated. Nonetheless, age or education did not significantly influence their preference for equal rationing. This non-significance may reflect the sample limitations. The orientations and background characteristics explained less than 20% of the variance in the preferences, indicating substantial unexplained variance. These limitations necessitate future research to maximize objectivity and representation. Future research should use stratified random sampling to represent China and the global population. To improve objectivity, researchers should survey informed individuals, such as experts and scientists, who are knowledgeable about public healthcare rationing, equity, and SOGIE. Future research can also juxtapose public and expert opinions to offer diverse views for policymaking. Furthermore, qualitative methods can help clarify public preferences. 

### 4.2. Implications

Considering the democratic value of public opinion, equal public healthcare rationing across SOGIE identities is preferable for achieving rational equity. This form of equity reflects a strong alignment with equality orientation and the rejection of self-interest. In contrast, universalist public healthcare rationing, which does not consider or affirm gender identity, is less preferable because of the possibility of deviation from equality and personal contribution principles. Nevertheless, universalist rationing is suitable because of its significant basis in public interest. Conversely, unequal rationing is the least preferred option because of its inconsistency with equity orientation and public interest and its susceptibility to self-interest bias.

## 5. Conclusions

According to the rationality model, equal public healthcare rationing for people with diverse SOGIE is preferable when equity orientations and public interest are at a maximum and self-interest is at a minimum. This projection rests on the responses of relatively young and more highly educated Chinese people. The preference is for affirmative rather than universalist rationing, which disregards gender identity. Hence, equal rationing to affirm gender identity is more rational than other approaches to achieve equity and public interest.

## Figures and Tables

**Table 1:** Means/percentages, standard errors, and standard deviations (*N* = 744).

Correlate	Scoring	*M*	*SE*	*SD*
Unequal rationing, now	0–100	32.8	1.6	42.4
Equal rationing, now	0–100	39.6	1.5	41.4
Universalist rationing, now	0–100	27.7	1.5	41.5
Equity orientation, past 3 months	0–100	56.9	1.0	24.6
Personal contribution orientation, recent month	0–100	55.8	1.0	25.0
Social contribution orientation, recent month	0–100	59.0	0.9	23.3
Equality orientation, recent month	0–100	57.9	1.0	24.4
Need orientation, recent month	0–100	58.8	1.0	24.2
Self-interest, recent month	0–100	57.9	1.0	24.1
Public interest, recent month	0–100	62.8	1.0	24.7
Social desirability, past 2 months	0–100	57.3	0.8	19.4
Acquiescence	0–100	46.3	0.8	21.9
Male	0, 100	40.5	2.0	49.1
Female	0, 100	59.0	2.0	49.2
Age	years	23.9	0.2	4.9
Education (0 = under college level; 33.3 = college; 66.7 = master’s; 100 = doctorate)	0–100	23.9	1.0	25.4
Heterosexual	0, 100	65.0	1.9	47.7
Irreligious	0, 100	80.4	1.6	39.7
Urban	0, 100	55.2	2.0	49.8
Married	0, 100	3.6	0.7	18.5
Via email	0, 100	19.6	1.6	39.8
Via a classmate	0, 100	25.6	1.8	43.7
Via social media	0, 100	30.4	1.8	46.1
Via a college forum	0, 100	24.2	1.7	42.8

**Table 2:** Standardized regression coefficients.

Predictor	Universalist Rationing	Equal Rationing	Unequal Rationing
Female	−0.038	0.032	0.006
Age	−0.070	0.073	−0.005
Education	0.099 *	0.015	−0.108 *
Irreligious	0.005	0.058	−0.059
Heterosexual	0.042	−0.021	−0.022
Married	0.180 ***	−0.094 *	−0.081
Urban	0.123 ***	−0.111 **	−0.010
College forum	−0.104 **	0.088 *	0.014
*R^2^*	0.078 ***	0.034	0.033 **
Acquiescence	−0.307 ***	0.238 ***	0.069
Social desirability	0.127 ***	−0.026	−0.015
Equity orientation	0.038	−0.023	−0.099 **
*R^2^*	0.158 ***	0.082 ***	0.042 ***
Personal contribution orientation	−0.155 *	0.036	0.117
Social contribution orientation	−0.040	0.038	0.002
Equality orientation	−0.119 *	0.110 *	0.009
Need orientation	0.035	−0.002	−0.033
Self-interest	−0.016	−0.198 *	0.209 *
Public interest	0.348 ***	−0.047	−0.295 ***
*R^2^*	0.199 ***	0.095 ***	0.076 ***
Δ*R*	0.202 ***	0.114	0.184 ***

VIF < 5.8, * *p* < 0.05. ** *p* < 0.01. *** *p* < 0.001.

**Table 3:** Projected means and standard errors at maximum rationality.

Rationality		Universalist Rationing	Equal Rationing	Unequal Rationing
Substantive + Public–instrumental	Mean	22.9	71.5	5.6
	Standard error	5.6	5.9	6.1

*Note.* Social desirability is at 0, and all other predictors are at averages.

## Data Availability

The raw data supporting the conclusions of this article will be made available by the authors on request.
